# ‘People should be allowed to do what they like’: Autistic adults’ views and experiences of stimming

**DOI:** 10.1177/1362361319829628

**Published:** 2019-02-28

**Authors:** Steven K Kapp, Robyn Steward, Laura Crane, Daisy Elliott, Chris Elphick, Elizabeth Pellicano, Ginny Russell

**Affiliations:** 1 University of Exeter, UK; 2 University College London, UK; 3 Macquarie University, Australia

**Keywords:** adults, autism, neurodiversity, repetitive behaviour, repetitive movements, self-stimulatory behaviour, stereotypies, stimming

## Abstract

‘Stereotyped or repetitive motor movements’ are characterised as core features in the diagnosis of autism, yet many autistic adults (and the neurodiversity movement) have reclaimed them as ‘stimming’. Supported by a growing body of scientific research, autistic adults argue that these behaviours may serve as useful coping mechanisms, yet little research has examined stimming from the perspective of autistic adults. Through interviews and focus groups, we asked 32 autistic adults to share their perceptions and experiences of stimming, including the reasons they stim, any value doing so may hold for them and their perceptions of others’ reactions to stimming. Using thematic analysis, we identified two themes: stimming as (1) a self-regulatory mechanism and (2) lacking in social acceptance, but can become accepted through understanding. Autistic adults highlighted the importance of stimming as an adaptive mechanism that helps them to soothe or communicate intense emotions or thoughts and thus objected to treatment that aims to eliminate the behaviour.

Research suggests that non-autistic people often misunderstand the behaviour of autistic people (Faso, Sasson, & Pinkham, 2015; Sheppard, Pillai, Wong, Ropar, & Mitchell, 2016), likely contributing to autistic people’s socio-communicative challenges. This particularly applies to ‘motor stereotypies’ such as ‘hand or finger flapping’ or ‘complex whole-body movements’ (American Psychiatric Association (APA), 2013). Since the initial accounts of autism (Asperger, 1944/1991; Kanner, 1943), these behaviours have been considered as self-stimulatory acts that shut out external stimuli and interfere with the person’s (and others’) focus (Lilley, in press). In addition, treatments to control (i.e. to eliminate, modify or reduce) ‘motor stereotypies’ remain popular clinically and in research (Lanovaz, Robertson, Soerono, & Watkins, 2013; Lilley, in press). Autistic adults have led resistance to these efforts (Lilley, in press), reclaiming ‘self-stimulatory behaviour’ as ‘stimming’ (e.g. Nolan & McBride, 2015). As autistic adults may understand one another better than non-autistic people understand them (Gernsbacher, Stevenson, & Dern, 2017; Gillespie-Lynch, Kapp, Brooks, Pickens, & Schwartzman, 2017; Komeda, 2015; Milton, 2014), this study sought to examine autistic adults’ perceptions and experiences of stimming.

Theoretical perspectives suggest that stimming has a sensorimotor basis. Delacato (1974) theorised that excessive, insufficient and inefficient sensory processing causes all autistic behaviours (grouped into ‘sensoryisms’), producing stimming as a controllable response. Similarly, Ornitz (1974) and Ornitz and Ritvo (1968) described autism as a syndrome of perceptual inconstancy, with motor output (like stimming) needed to modulate inconsistent sensory input. In support of this theory, autistic people have shown high behavioural and neurological variability to the same basic stimuli over time (Colbert, Koegler, & Markham, 1959; Haigh, 2018). Furthermore, more recent theories have suggested that stimming may provide familiar and reliable self-generated feedback in response to difficulties with unpredictable, overwhelming and novel circumstances (e.g. Lawson, Rees, & Friston, 2014; Pellicano & Burr, 2012). As such, stimming may provide not only relief from excessive sensory stimulation, but also emotional excitation such as anxiety (Leekam, Prior, & Uljarevic, 2011). Consistent with these suggestions, autistic adults report that stimming provides a soothing rhythm that helps them cope with distorted or overstimulating perception and resultant distress (Davidson, 2010) and can help manage uncertainty and anxiety (e.g. Joyce, Honey, Leekam, Barrett, & Rodgers, 2017).

Reflecting the aims of popular interventions, language surrounding the topic of stimming is often pejorative (Jaswal & Ahktar, 2018). Researchers sometimes assume that stimming falls within voluntary control and has asocial or antisocial motivations (Jaswal & Ahktar, 2018; Lilley, in press). For example, a prominent review of repetitive behaviours in autistic people attributed the onset of stimming to a ‘self-imposed restricted environment’ (Leekam et al., 2011, p. 577). Stimming has become so associated with autism that some scientists and clinicians use the term ‘stims’ interchangeably with ‘autistic behaviour’ (Donnellan, Hill, & Leary, 2013). Furthermore, therapies continue to treat stimming despite lacking strong evidence of efficacy or ethics (Jaswal & Akhtar, 2018; Lilley, in press). While researchers increasingly acknowledge limitations in the understanding of, and interventions for, stimming (e.g. Harrop, 2015; Patterson, Smith, & Jelen, 2010), treatments may remain popular, in part because many parents regard it as noticeable and stigmatising (Kinnear, Link, Ballan, & Fischbach, 2016).

Autistic people have become increasingly mobilised and vocal in defence of stimming. Autism rights or neurodiversity activists believe that stims may serve as coping mechanisms, thus opposing attempts to eliminate non-injurious forms of stimming (e.g. Orsini & Smith, 2010). They decry practices such as ‘quiet hands’ (which teaches the suppression of hand flapping), instead using ‘loud hands’ as a metaphor both for using such non-verbal behaviour to communicate and for cultural resistance more broadly (Bascom, 2012). In addition, autistic scholar-activists denounce attempts to reduce their bodily autonomy (Nolan & McBride, 2015; Richter, 2017) and declarations of their stimming as unacceptable or as necessarily involuntary (Yergeau, 2016).

This research, co-produced by autistic self-advocates alongside researchers who do not identify as autistic, sought to further understand the issue of stimming from autistic adults’ perspectives. It builds on the only empirical study, at least to our knowledge, to have directly elicited autistic adults’ views about this topic. Steward (2015), in an online survey study of 100 autistic adults, highlighted a wide range of reasons for stimming, including a coping mechanism to reduce anxiety (72%) or overstimulation (57%), or to calm down (69%). Furthermore, 80% of survey respondents reported that they generally or sometimes enjoyed stimming (with another 11% indicating that their enjoyment depended on the particular stim), yet 72% had been told not to do it.

Here, we sought to extend Steward’s (2015) work by eliciting autistic adults’ views using in-depth semi-structured interviews and focus groups. Specifically, we aimed to examine autistic adults’ (1) understanding of repetitive or ‘stimming’ behaviours, (2) perceptions of the reasons underpinning such behaviours (i.e. why they stim) and (3) views on the value, if any, of such behaviours.

## Method

### Participants

A total of 31 autistic adults (20 male, 10 female and 1 non-binary), between the ages of 21 and 56 years, participated in the study. Of them, 19 took part in interviews and 12 took part in focus groups (see Table 1). Recruitment took place in two regions of England (the Southwest and London). To sample autistic adults with wide-ranging needs, the Southwest team recruited interview participants through residential homes specialising in housing autistic adults, a training centre for autistic adults and existing networks. Recruitment for focus groups took place through existing networks of both research teams.

Participants had an independent clinical diagnosis of an autism spectrum condition, according to International Classification of Diseases, Tenth Revision (ICD-10; World Health Organization, 1992) or *Diagnostic and Statistical Manual of Mental Disorders* (5th ed.; DSM-5) criteria (APA, 2013). Diagnoses included Asperger’s syndrome (*n* = 16), autism (*n* = 9) and autism spectrum disorder (*n* = 6). In total, 21 participants received their diagnosis in adulthood and 10 in childhood. Of the sample, 16 were currently unemployed (including one looking for work), 10 were in some form of employment (including three in voluntary employment) and 5 were students.

**Table 1.** Participant information.

Participant	Gender	Age range	Focus group or interview (in person unless stated otherwise)
Rebecca	F	21–30	Focus group
Sinead	F	41–50	Focus group
Fiona	F	31–40	Focus group
Greg	M	41–50	Focus group
Layla	F	21–30	Focus group
Ian	M	41–50	Focus group
Alex	Non-binary	31–40	Focus group
Philip	M	21–30	Focus group
Ethan	M	21–30	Focus group
Martin	M	31–40	Focus group
Roger	M	21–30	Focus group
Clive	M	31–40	Focus group
Anthony	M	21–30	Interview (e-mail, instant messaging)
Alana	F	41–50	Interview (e-mail)
Jared	M	21–30	Interview
Miles	M	31–40	Interview
William	M	31–40	Interview
Claire	F	31–40	Interview
Joseph	M	41–50	Interview
Rueben	M	41–50	Interview
Rose	F	51–56	Interview
Luke	M	21–30	Interview
Sam	M	41–50	Interview
Roman	M	51–56	Interview
Sally	F	21–30	Interview
Abby	F	21–30	Interview
Lucy	F	41–50	Interview
Michael	M	41–50	Interview
Victor	M	51–56	Interview
Max	M	31–40	Interview
Ed	M	21–30	Interview

### Interview and focus group protocols

The individual (more detailed) interviews and (lengthier) focus groups provided complementary approaches to triangulate data on stimming. Ethical approval was granted by the University of Exeter’s Social Studies and International Studies’ College Ethics Committee (201516-066) and UCL Institute of Education’s Research Ethics Committee (REC 924). Participants provided written, informed consent prior to taking part. Interviews and focus groups were digitally recorded and transcribed verbatim, with quotations presented verbatim in section ‘Results’. To preserve anonymity, pseudonyms are used throughout the article.

#### Interviews

In total, 19 autistic adults took part in individual semi-structured interviews, conducted by G.R., D.E. and C.E.; of these, 17 interviews took place in person (in a dedicated, quiet room), 1 took place by e-mail and 1 took place by both e-mail and instant messaging. Participants had the option to have a parent/carer present during the interview, and five chose to exercise it (all in person). Questions about stimming (which took approximately 15 min) took place as part of a larger interview (which took approximately an hour), which also included questions about their strengths (that form the basis of a separate manuscript; see Table 2 for the interview topic guide).

**Table 2.** Interview schedule used in interviews and focus groups, with main questions and prompts.

Key question	Prompts
Do you do any stims, or repetitive movements?	Which ones do you do? Which ones do you do the most? What kind of movements do you class as stimming? What do you do when you stim? Do you use something to stim with? How often do you stim? How long do you stim for?
What triggers your stims?	Can you give me an example of any situations that might cause you to stim? When do you stim? What is the reason you do them, do you think?
Is it helpful/useful?	Does stimming make you feel better? In what way?
What would happen if you could not stim?	Has anyone ever told you not to stim? How would/does it make you feel? What would/do you do and not do? Why?

#### Focus groups

Following the interviews, we invited new participants to take part in one of two face-to-face focus groups on stimming (*n* = 6 per group), lasting approximately 60–90 min, in a location convenient for participants. Groups were led by facilitators (G.R., R.S. and S.K.K. for one group; R.S. and E.P. for the other), who, at key moments during the discussion, fed the main points back to the group to confirm their interpretation of key messages. They also encouraged all participants to contribute to discussion. Focus groups were conducted according to a semi-structured interview schedule, with sticky note activities used to aid discussion and enable everyone to contribute. The first sticky note activity involved asking participants to note examples of ‘stimming’ or ‘repetitive movements’, which were subsequently discussed as a group (see Table 2 for questions). A final sticky note activity instructed participants to write down potential causes of their stims. Other sticky note activities and discussion also centred on fidgeting (in comparison to stimming), the focus of a separate paper.

### Data analysis

We did not differentiate data collected from interviews and focus groups in our analysis, as per other qualitative research studies. Data were analysed thematically, following Braun and Clarke (2006). We adopted an inductive approach to data analysis, an experiential orientation to data and a critical realist theoretical perspective (c.f. Braun & Clarke, 2012), to systematically examine adults’ subjective accounts of their meanings and experiences. Quotations were used to illustrate identified themes. Motivated by the literature (especially from autistic adults’ perspectives) suggesting that diagnostic symptoms of repetitive motor movements sometimes function as a coping mechanism, we coded the data deductively with regard to the meaning and forms of, the reasons for and the utility of, stimming. We also analysed data inductively for any other identified patterns.

The team read the interview and focus group transcripts, with two authors (S.K.K. and G.R.) immersing themselves in the data by reading them twice and taking notes on striking and recurring observations. G.R. developed a coding framework with suggestions from the study team, and D.E. used it to code the data. S.K.K. read all the data extracts (organised by code) and fitted them into larger categories, giving each a new title and a written summary. S.K.K. then generated a draft thematic map, reviewed by G.R. and D.E., which was subsequently revised, before writing the analysis and sending it to the study team for comment and discussion. The study team’s training in social science (psychology for S.K.K., G.R., L.C. and E.P., and sociology for S.K.K., G.R., C.E. and D.E.) and positionalities as autistic researchers (S.K.K. and R.S.) informed our analysis.

## Results

**Figure 1.**
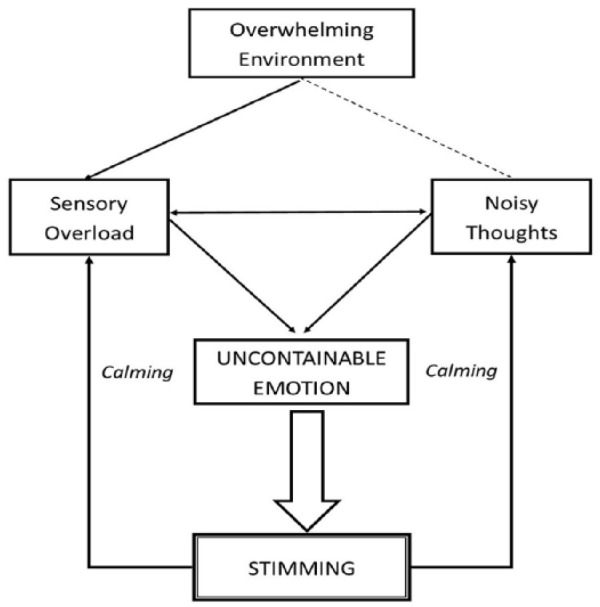
Stimming as a self-regulatory mechanism.

**Figure 2.**
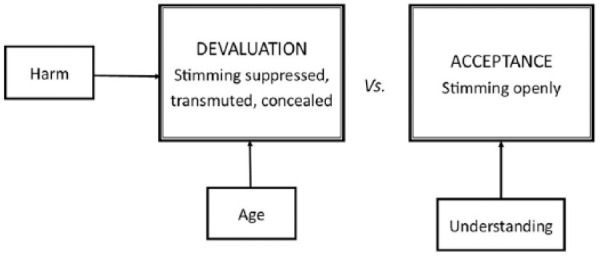
(De)stigmatisation of stimming.

Participants discussed what stimming comprised and how it affected their lives. In most cases, they described stimming as a series of repetitive movements such as hand flapping, rocking and flicking (see Theme 1). Two major themes were identified: ‘Stimming as a self-regulatory mechanism’ (see Figure 1) and ‘(De)stigmatisation of stimming’ (see Figure 2), each comprising several subthemes (themes and subthemes were robust to both interviews and focus groups).

### Theme 1: stimming as a self-regulatory mechanism

Stimming was identified as a repetitive, usually rhythmic behaviour that was commonly expressed through body movements (variously described as hand flapping, finger flicking, hair pulling or pinching, feet flexing, spinning, necklace playing) but also vocalisations (e.g. muttering, grunting, stuttering, whistling, singing). Many participants said they experienced it as involuntary and unconscious, at least at the beginning of the behaviour. Although many described stimming as automatic and uncontrollable, no participants consistently and inherently disliked their stims (as opposed to their social consequences). Indeed, most participants described stimming itself as comfortable or calming, suggesting a self-regulatory function (which some participants explicitly identified). The accounts of our participants suggested that stimming created a feedback loop that regulated excess emotion and was self-perpetuated because of the soothing comfort or control afforded by the behaviour.

Our analysis revealed four subthemes, including overwhelming environment, sensory overload, noisy thoughts and uncontainable emotion. These were interrelated: an overwhelming environment was described as producing sensory overload and sometimes contributing to noisy thoughts. Externally generated senses (like loud noises and sudden movements from children during lunchtimes at school) or internally generated thoughts (such as nagging worries about work) could independently or together cause excessive uncontainable emotions (such as anxiety), resulting in the self-regulatory mechanism of rhythmic behaviour known as stimming. A schematic illustration of this process is shown in Figure 1.

As one participant (Luke) explained, the regulatory aspect of stimming worked through attending to a single point of focus over which one had control, to self-regulate by blocking or reducing excessive input. He suggested that the cause of this either comes externally (through sensory bombardment) or internally (through a flood of thoughts). He described how he controlled stimulation by rotating his wrist. The rhythm enabled him to self-regulate his mind and body according to the timing of the predictable movement:

it helps you talk to yourself at a rhythmical pace, so when I’m doing this I can sort of think in the rhythm that I’m moving my hand … which is very helpful because it means like when you’ve got your internal monologue it doesn’t all come in at once and you find yourself sort of shouting at yourself in your head to get everything done.

In turn, this self-stimulating motion calmed the body ‘in time with the pendulum’: ‘it sort of metronomes everything in your body to sort of go at that speed … So it just sort of helps quell everything, because you’re at the same rhythm with everything’.

Below we report our four subthemes in the context of stimming as a regulatory mechanism.

#### Subthemes 1 and 2: overwhelming environment and sensory overload

Very often, participants described external causes of stimming such as confusing, unpredictable, overwhelming environments. This inextricably led to excessive sensory stimulation, contributing to stressful emotional states. They described sensory hypersensitivities as relating to interpersonal difficulties that may generate the need for a coping mechanism like stimming.

Returning to Luke, he described the long hours of working to support people with ‘so-called severe autism’ as producing ‘a lot of sensory information’ that led him to stim ‘quite a lot’, especially at mealtimes with the service users (while at work). The following example illustrates the demands of simultaneous multisensory processing (visual, gustatory (taste) and auditory) amid a context of long work hours and social responsibility for others’ well-being:

I need to be looking out for them, but I’ve got lots of sort of taste senses coming in and I’ve got lots of sound stuff, I was like tuning in to conversations or whatever and sort of keeping an eye out in case somebody comes up the stairs wanting their meal now, wanting supporting, whatever.

Thus, he usually stimmed when in a room to himself but sometimes could not help the hand not used for eating ‘just rolling itself around like in a sort of circular motion’, forcing him to explain his behaviour and request more breaks.

The social acceptability of stimming in the current context (see Theme 2), and the extent to which individuals had awareness of and voluntary control over the action, influenced whether stimming took place. Task demands and physical and mental states (e.g. energy levels and emotional well-being) also reportedly influenced stimming. These factors affected how people used stimming to engage with or withdraw from the environment. For example, Max was ‘slightly closing my eyes a fair bit during this interview’, which he described as a stim that helped him concentrate:

The eye close is to cut off additional stimuli so I don’t get tired, or sometimes when I can particularly obsessively focus on the one thing that needs to happen. So contrary to what would appear common sense, I close my eyes quite a few times during dances which I didn’t understand because I needed to have the other person lead me more than trying to see what’s happening.

None of the participants described instances of too little sensory input or sensory hyposensitivities as a causal factor for stimming or generally, but many attributed stimming to what Victor called ‘sensory overload’.

#### Subtheme 3: noisy thoughts

Participants described dysregulated, excessive or distracting thoughts that led to stimming, sometimes associated with specific stims. For example, Alex described songs playing in his head that triggered distressing memories, leading to quick rolls forward and backward on his wheelchair and repeating a word associated with the memory.

These thoughts were often triggered by their surroundings and interacted with sensory stimulation. Several of their experiences suggested that the noisy thoughts that led to stimming were less preventable than sensory-based causes. This was due to greater ability to modify their environment than their mental state. For example, Rose described hypersensitivity to noise that threatened to raise her heartbeat but explained that she could avoid this, for example, by sitting in her car during her lunch break for ‘peace and quiet’. In doing this, she stated, ‘I’ve got the environment how I want it and my stress, on the whole now, is more self-inflicted, like having an assignment to do’, which triggered stims like leg jiggling, finger tapping and body rocking.

#### Subtheme 4: uncontainable emotion

Most participants identified an abundance of at least one emotional state as the most proximal cause of stimming. No accounts contradicted this pathway. The descriptor *uncontainable* refers to the magnitude of the emotional state, causing its expression through stimming behaviour. Stimming served a communicative, as well as a regulatory, function. Some participants described stimming in response to positive emotional states (e.g. excitement) and others in response to negative emotional states (e.g. anxiety). Valence of emotions (positive or negative) varied but the potency of the emotion itself emerged as a consistent pattern, with stimming calming a state of hyperarousal. As Rebecca explained, ‘[s]timming is just a release of any high emotion, so really anxious, really agitated, really happy, really excited, just any high emotion, that’s when I stim’. Stimming appeared to function to calm.

For some participants, particular stims always responded to a particular emotion, which meant that the behaviours may have effectively communicated the person’s feelings (and those close to them may come to understand this meaning). For example, with regard to head-picking, Sinead explained ‘my son and husband get really upset if I’m doing that because it means that I’m really emotional and stressed’. Emotional valence may shape the specific form that the same general behaviour takes. For example, several participants explained that they flap their hands both when excited or happy as well as when distressed, and two (Alana and Ethan) detailed that hand flapping due to positive states involves hands open and arms out in a waving-like motion, unlike hands and arms kept further down towards oneself (when in a more negative state).

Although usually described as instinctive and reactive, according to several participants, stimming could also be under conscious control and used actively to prevent emotional dysregulation. Sally said she learned about stimming’s soothing benefits through resources (e.g. YouTube videos) that help autistic people stim:

And I started kind of incorporating it more in my life, and it actually managed to help me stave off some panic attacks. For example, I never used to wave my hands that much, but I’ve started doing it more and it actually helps, like if I’m in a crowded elevator or something.

### Theme 2: (de)stigmatisation of stimming

The second theme concerned (1) the negative reactions that people perceived when stimming and (2) destigmatisation through acceptance based on social understanding of their stims. Participants described feeling a variety of resentful emotions when told by others to stop stimming, including anger, nervousness, frustration, belittlement, shame and confusion. They expressed that others might feel annoyed, stressed or alarmed by their stims, and stated that observers might view them as strange, aggressive, sad, ridiculous or childish. Many wished to avoid drawing negative attention and, in response to feeling marginalised, attempted to suppress their stims in public. They also reported stimming when alone, for this reason.

Other participants reported transmuting stims into a more socially acceptable form that provided similar feedback. For example, Ethan replaced arm stims with dancing, shaking hands, tennis, chess and sailing. Alternatively, participants tried concealing stimming from view. Repression of stimming happened more as a function of whether people said they felt understood. Participants encountered accepting attitudes from others more often in private than in public. This was because of greater understanding (through others’ familiarity with them or their knowledge of autism and the reasons for stimming). Several only stimmed freely when they had total privacy (i.e. on their own) or among selected (accepting) family or friends.

Figure 2 illustrates the conceptual map developed to try to represent both dimensions of (de)stigmatisation that participants described. Influences could act either to lead to devaluation or increase acceptance of stimming. Subtheme 1 includes promoters of devaluation and Subtheme 2 concerns acceptance, with the path mediated through understanding.

#### Subtheme 1: devaluation

Several participants internalised the stigmatisation of stimming, with ambivalent attitudes despite recognising the utility of their stims. Rose reported hiding stims (e.g. stimming on her leg rather than on her desk at university) and ‘if I thought anyone could see what I was doing, I could have stopped it’. She taught her students to do the same: ‘I’d try and get students not to display’ if ‘they didn’t want to be seen as different’, telling them ‘You’re disturbing everyone, you’re causing attention’ for actions like jiggling their legs and fidgeting with their watch. Nevertheless, she admitted stimming ‘helps me keep me calm … it cuts down what is going on around, it helps me focus’ and that suppressing her stims made her feel ‘just sort of more on edge’. Pointedly, Rose critiqued intervention based in ‘ABA’ (applied behavioural analysis) in which ‘they basically condition them like Pavlov’s dogs to stop stimming’, remarking,

to me it was abuse, because stopping those children stimming when they’re trying to calm themselves down or cope with a situation, because even if they manage all the environment around them, there might be situations that they find stressful, and if they haven’t got the ability to calm them down, then they could be relying on other people for the rest of their lives or have a breakdown …

In addition to stims that made them feel devalued for appearing ‘weird’, participants described stims that caused (unintentional) harm to themselves or others. They largely recognised that others would not accept harmful stimming and tried to suppress stimming that caused self-harm.

##### Harm

Participants gave several examples of stimming behaviours that caused physical harm to themselves (with no evidence of intentional self-injury), which they did not find helpful, regardless of social context. For example, Max explained that he repeatedly pressed his fingers together when anxious for sensory feedback, but did not find it helpful ‘because you can get into a loop and you can start really making your fingers leathery if you’re not careful’. Nevertheless, all participants gave examples of stims they found inherently helpful, although they did not necessarily like it when others noticed their engagement in the behaviour.

Participants described the annoyance and distraction their stimming may cause others. Greg described obliviously clicking his nails at home, including near the ear of his wife, who has ‘good hearing’ and ‘really hates that’. Similarly, Sinead described inadvertently bringing pain to her husband: ‘apparently, I did pinch around his nails or something like that, I don’t know’. Sinead’s husband brought the behaviour to her attention when he became ‘really sore … [T]hat’s a bit distressing to find out you’re causing harm to somebody without realising you’re doing that’. The exception came from Sam, who stimmed to antagonise support staff at home. Other participants talked about having struggled to interpret their stimming as harmful even when others express irritation. They discussed disruption as avoidable if others did not focus on their stimming activity, or if others became more open-minded. Several participants made declarative statements that their stims were not ‘affecting’ or ‘hurting’ anyone, or ‘doing any harm’ and so stated they should be accepted. Similarly, Claire said she could understand attempts by others to stop stimming ‘if it’s harmful to somebody else or it’s annoying to somebody else, but if it’s absolutely got no bearing on another person whatsoever I think people should be allowed to do what they like’.

##### Age

Participants reported that stimming became less socially acceptable as one got older. Layla defined stimming in this way: ‘I think of stims as the kind of behaviours that either autistic people [do] or what you do when you’re a young child and that you normally grow out of’. Several participants offered compatible narratives from their own experience, in that they stimmed happily as young children but by secondary school (11–16 years) norms changed, and they hid or transmuted stimming once aware of negative judgement. This may also reflect greater self-awareness: ‘[P]robably the people that saw me doing it [hand flapping], peers that might have judged me badly, but until I got to secondary school I didn’t realise that other people were judging me badly for it’ (Clive).

Participants’ comparisons between childhood and adulthood referred to how stigmatisation of stimming infantilises autistic people, who may fear they come across as ‘immature’ (Roger). When told not to stim as an adult, Clive said he feels ‘belittled’, as though he is ‘five’ being told off for ‘genuine misbehaviour’. ‘[I]t makes me feel that age again … I shouldn’t feel like I’m in reception class again learning basic things’. Sinead flashbacked to the highs of stimming she used to freely enjoy:

I remember as a child spinning all the time and loving spinning and loving swinging and feeling that movement all the time, but then I also realised that there was a point where it wasn’t acceptable to be spinning anymore … so it actually still feels glorious if there’s nobody around and I can skip or I can spin and it’s like I’m breaking the rules.

#### Subtheme 2: acceptance

Acceptance enabled participants the freedom to stim openly. Anthony said no one had ever told him not to stim, explaining, ‘I’m in a very autism-accepting environment and gr[e]w up in a special school’. This attitude liberated him to stim (e.g. hand flapping, foot shaking, rocking) ‘as often as I’m excited or anxious’ even though his stimming included the extreme dysregulation of a ‘meltdown phase’ (in which he rocked, but ‘standing and rocking, not sitting’).

##### Promoting acceptance through understanding

Understanding held the key to acceptance of non-harmful stimming in autistic adults, according to our analysis. Greg’s wife used to work for an autism advocacy organisation, so with reference to his stimming, ‘because she understands it, she knows why I’m doing it … she lets me get on with it most of the time …’ She further practised her professional skills by helping him to write an e-mail explaining his autism and stimming in the workplace, which achieved its goal and boosted his productivity as he worried less about stimming.

Other participants did not fare as well with their family. For example, Rebecca’s aunt, uncle and grandmother attempted to stop her hand flapping both verbally and physically. She said that she felt ‘[a]ngry that they’ve been told a thousand times why I do it, the reason behind it, that it’s not affecting anyone’. In response to another focus group member’s comment, ‘It’s probably because they don’t understand’, she remarked, ‘But, they should because they’re my family’.

## Discussion

Through interviews and focus groups, autistic adults with various support needs shared remarkably similar perceptions of repetitive, stereotyped behaviours known as ‘stimming’. A robust pattern emerged of stimming as a self-regulatory mechanism, which acted to create a calming feedback loop. According to participants, intense emotions could have either a positive (e.g. happy or excited) or a negative (e.g. anxious or distressed) valence, and the corresponding stims could have a different manifestation depending on valence. Stimming was therefore reported to be a useful behaviour, serving to contain or control excess emotion, and the social acceptability of stimming was perceived to depend on a number of cultural factors including age, familiarity and understanding of autism. Although no scientific literature (to our knowledge) directly examines a reported communicative function of stimming, our findings support accounts of autistic activists, which suggest that the language of stimming may assist with learning to recognise the inner emotional states of autistic adults (Bascom, 2012; Lindsmith, 2014; Schaber, 2014).

None of the participants reported their stimming behaviours to be thoroughly detrimental, although they regularly encountered negative social judgements that made them feel self-conscious about stimming around others. Indeed, participants commonly responded to the negative attention their stims attracted by suppressing stimming behaviours: transmuting them into a more socially acceptable form that provided similar feedback, or concealing them away from others’ view. Not all participants reported voluntary control over their stimming, but even those who said they could suppress their stims described depleting, effortful costs. In parallel, autistic bloggers have described strains of suppressing stimming, such as invoking Baumeister’s (2002) ego depletion model of self-control (which describes self-control as a limited resource like a muscle; Kim, 2013) and describing how their performance suffered after transitioning to a new and more independent context (e.g. going to university; Gross, 2011) while they suppressed their urge to stim.

Participants professed no desire for self-injurious stims and largely wished to avoid stimming in ways harmful to others. Although the sample was recruited from the general community, rather than a subsection of activists (and thus seemed to vary greatly in their identities regarding autism), this finding is consistent with the neurodiversity movement’s opposition to eliminating all stimming with the exception of behaviours harmful to inclusion and quality of life (c.f. Ne’eman, 2010; Robertson, 2010). Our participants’ perspective is consistent with the practice of making stims a target of treatment only when injurious, which (according to the DSM-5) would call for a co-occurring diagnosis of stereotypic movement disorder (APA, 2013). Seeking to extinguish functional stimming would violate the medical ethics of the principle to ‘do no harm’ (c.f. Nicolaidis, 2012).

Participants described stimming as helping to calm or soothe overwhelming sensations or emotions, which is consistent with self-reports of autistic individuals (e.g. Joyce et al., 2017; Steward, 2015). We also extend the literature by providing accounts that suggest stimming helps with concentration and learning. Stimming seemed to give autistic people a mechanism of behavioural control to self-regulate a state of emotional hyperarousal, amid a bombardment of overwhelming sensations or thoughts, consistent with classical and modern theories of autism (e.g. Delacato, 1974; Ornitz, 1974; Pellicano & Burr, 2012). Self-reported experiences may shed light on the empirically supported theory of autism as a syndrome of perceptual inconstancy (Ornitz & Ritvo, 1968), as participants may not perceive their sensory and social surroundings as intensely when stimming. Moreover, stimming may help process dynamic, simultaneous and unpredictable sensations (such as in the care home where Luke worked), which may dovetail with theories that seek to explain why autistic people often struggle with sensory processing and novelty (e.g. Pellicano & Burr, 2012).

Participants in the current sample reported that others’ understanding held the key to social acceptance of their stims. This may apply particularly as they age, because they reported that negative reactions became more common as they grew up. Behaviours like rocking and hand flapping typify infancy but become less common as children get older (Leekam et al., 2011). Such stims may function to help infants transition to volitional motor activity (Thelen, 1979), yet many autistic people struggle with the initiation and execution of intentional skilled movements into adulthood (Biscaldi et al., 2014; Wild, Poliakoff, Jerrison, & Gowen, 2012). Such evidence links with theories of autism as affecting rhythms (Amos, 2013; Tordjman et al., 2015) and Luke’s description of stimming as coordinating thoughts and activity at the pace of his movements to ‘quell everything, because you’re at the same rhythm with everything’. Therefore, stimming may have underappreciated benefits in assisting autistic people (even adults) with motor control.

Our findings overlap with the perspectives of occupational therapy and sensory integration theory that stimming may result from sensory dysregulation (Lilley, in press; Miller, Anzalone, Lane, Cermak, & Osten, 2007) but do not necessarily suggest a similar route for intervention. Instead, the views and experiences of participants suggest that modifying the environment (so it does not provoke stimming) and promoting social acceptance are key. Potentially, this could address the underlying processing difficulties experienced by our sample, but not seek to reduce non-injurious stimming for its own sake. Ultimately, our findings make us assess repetitive motor behaviours in a different light to that cast by medical texts (Jaswal & Akhtar, 2018; Lilley, in press). The autism field would be best placed to take a more nuanced look at why autistic people perform repetitive motor behaviours so frequently to inform subsequent revisions of diagnostic criteria. Rather than aiming to obliviate all stims, perhaps support for interventions that aid non-harmful stimming and reduce prejudice is the way forward.

Potentially, other people’s imitation of their stims may facilitate autistic people’s self-other awareness (see Kapp, 2016, p. 70) and help bridge the difficulties autistic and non-autistic people share in understanding one another, which Milton (2012) terms the ‘double empathy problem’. Interventions should facilitate true reciprocity that helps *non-*autistic people understand and respect stimming (Gernsbacher, 2006; Pellicano, 2013).

### Strengths and limitations

The study is the first in-depth examination of stimming from the perspective of autistic people. While positive accounts of stimming mostly stem from self-selected, highly articulate and self-aware segments of the autistic community, such as writers of autobiographies (e.g. Davidson, 2010), participants in advocacy organisations (e.g. Savarese, 2010) and scholars (Nolan & McBride, 2015; Richter, 2017), this study actively recruited predominantly non-activist participants largely in their own settings (e.g. group supported living accommodation, their parents’ house, a training centre). Past studies emphasise anecdotes from North America, where behavioural therapies aimed at reducing stimming appear more common (e.g. Orsini & Smith, 2010). Recruiting participants from their communities in a country (the United Kingdom) where stimming might be more socially acceptable than in the United States (*BBC News*, 2013), we found stigmatisation and misunderstandings still commonly reported by autistic adults. E-mail and instant messaging (in addition to in-person) options for interviews enhanced the geographical diversity of the sample within the United Kingdom. While it is common for interview and focus group data to be analysed together (e.g. Bauer, Yang, & Austin, 2004; Eysenbach & Köhler, 2002), it is not known whether differences in our data collection methods affected the results. Furthermore, the absence of systematic data on the participants’ clinical functioning and diagnoses, and the lack of participants with severe intellectual disabilities or minimal language, poses limitations. The study findings also may not transfer to children, non-speaking autistic people or autistic people living outside the United Kingdom.

## Conclusion and future directions

The results have implications for supporting autistic people. They suggest that many autistic adults agree with the neurodiversity movement’s opposition to eliminating all forms of stimming across all contexts (e.g. traditional uses of early intensive behavioural intervention) and desire for society to accept non-harmful forms of stimming (Bascom, 2012; Lilley, in press). Indeed, we found potential evidence of the spread of the movement through the report of teaching oneself how to stim via online resources. Our study suggests that carers, staff and autistic adults themselves may prevent the need for stimming in certain cases, such as environmental adjustments to reduce the risk of sensory overload. The point of intervention could therefore be shifted to the overwhelming environment rather than the autistic person themselves. Yet participants described stimming caused by uncontrollable thoughts as more difficult to prevent. Possibly, participants struggled to change their thoughts because of the ‘rigid thinking patterns’ characteristic of autistic people (APA, 2013); autistic adults often report low ability to reframe bothersome thoughts to prevent or alleviate distress (Samson, Huber, & Gross, 2012). Future researchers could investigate autistic people’s accounts regarding the supposed internal causes of their stims, including sensory (hyper)sensitivities, cognitive inflexibility and emotional dysregulation, and whether these should be addressed.

Other future directions include investigating the role of stimming and potentially related behaviours beyond autism. For example, one might compare ‘stimming’ in autistic people with ‘fidgeting’ in non-autistic people, as some scholars (e.g. Jaswal & Akhtar, 2018) and autistic advocates (e.g. Lindsmith, 2014) suggest that everyone stims as a coping mechanism. Research beyond autism suggests that children often do not regard their own or others’ repetitive behaviours as problematic (Harris, Mahone, & Singer, 2008) and that they may offer benefits (e.g. concentration for children with attention deficit hyperactivity disorder (ADHD); Hartanto, Krafft, Iosif, & Schweitzer, 2016). Greater understanding of such repetitive behaviours may, therefore, help elucidate appropriate support for a variety of people.

## Supplemental Material

AUT829628_Lay_Abstract – Supplemental material for ‘People should be allowed to do what they like’: Autistic adults’ views and experiences of stimming Supplemental material, AUT829628_Lay_Abstract for ‘People should be allowed to do what they like’: Autistic adults’ views and experiences of stimming by Steven K Kapp, Robyn Steward, Laura Crane, Daisy Elliott, Chris Elphick, Elizabeth Pellicano and Ginny Russell in Autism
