# Supplementary material for: ‘People should be allowed to do what they like’: Autistic adults’ views and experiences of stimming
Source: Autism. 2019 Feb 28;23(7):1782–92. doi: 10.1177/1362361319829628 (PMC6728747; doi:10.1177/1362361319829628)
Supplement: AUT829628_Lay_Abstract – Supplemental material for ‘People should be allowed to do what they like’: Autistic adults’ views and experiences of stimming [file AUT829628_Lay_Abstract.pdf]

Lay Abstract

autism

DOI: 10.1177/1362361319829628

‘People should be allowed to do what they like’: Autistic adults’ views and experiences of stimming

Steven K Kapp, Robyn Steward, Laura Crane, Daisy Elliott, Chris Elphick, Elizabeth Pellicano and Ginny Russell

‘Stereotyped or repetitive motor movements’, like finger-flicking, hand-flapping, body rocking, and spinning, are important in the diagnosis of autism. Yet many autistic adults (and the neurodiversity movement) have reclaimed these behaviours as ‘stimming’, arguing that these behaviours may serve as useful coping mechanisms. There is almost no research, however, examining stimming from the perspective of autistic adults. Through individual and group-based interviews, we asked 32 autistic adults to share their perceptions and experiences of stimming, including the reasons they stim, why they do so, and how others have tended to react to their stimming. Our results highlighted that autistic adults perceived stimming as self-regulatory. They described how these behaviours soothe or communicate overwhelming (positive or negative) emotions caused by intense sensations, feelings, or thoughts. Despite often having this calming effect, they reported feeling that these behaviours were not accepted by others, which often led to them trying to suppress their stimming. The exception to this pattern occurred when they felt that others understood why they stimmed, which encouraged participants to stim openly and therefore be themselves. Our participants therefore objected to treatment and interventions that aim to eliminate the behaviour. Instead, they wished for these behaviours to be recognised and accepted as behaviours that are functional in nature, helping them to cope with an often confusing and overwhelming world.
